# The anatomy of job polarisation in the UK

**DOI:** 10.1186/s12651-018-0242-z

**Published:** 2018-07-25

**Authors:** Andrea Salvatori

**Affiliations:** 1 OECD, Paris, France; 2 ISER, University of Essex, Colchester, UK; 3 IZA, Bonn, Germany

**Keywords:** Job polarisation, Occupational mobility, J21, J23, J24, O33

## Abstract

This paper studies the contribution of different skill groups to the polarisation of the UK labour market. We show that the large increase in graduate numbers contributed to the substantial reallocation of employment from middling to top occupations which is the main feature of the polarisation process in the UK over the past three decades. The increase in the number of immigrants, on the other hand, does not account for any particular aspect of the polarisation in the UK. Changes in the skill mix of the workforce account for most of the decline in routine employment across the occupational distribution, but within-group changes account for most of the decline in routine occupations in middling occupations. In addition, there is no clear indication of polarisation *within* all skill groups—a fact that previous literature has cited as evidence that technology drives the decline of middling occupations. These findings differ substantially from previous evidence on the US and cast doubts on the role of technology as the main driver of polarisation in the UK.

**Electronic supplementary material:**

The online version of this article (10.1186/s12651-018-0242-z) contains supplementary material, which is available to authorized users.

## Introduction

The increasing ability of technology to replace workers in performing easier-to-codify “routine” tasks has been singled out in the literature as the main driver of “job polarisation”, i.e. the decline in the share of mid-pay mid-skill jobs observed in several developed countries (Autor 2014; Goos et al. 2014). Recent contributions, however, have highlighted that the occupational wage patterns observed in many countries do not fit the predictions of the so-called routine-biased technology change (RBTC) hypothesis, igniting a debate among scholars and policy makers on the importance of technology in driving changes in the occupational structure.^1^

**Fig. 1:**
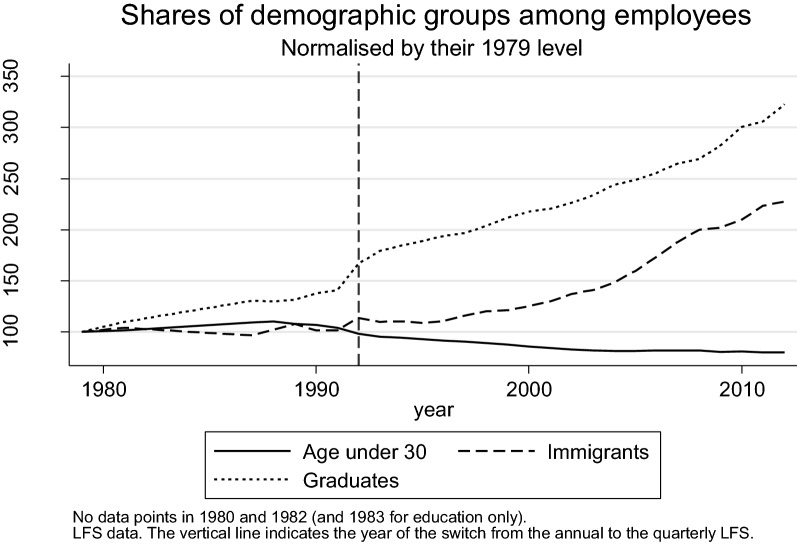
Changes in shares of skill and demographic groups between 1979 and 2012

This paper contributes to this debate by investigating the role of changes in the supply of different skill groups in explaining job polarisation in the UK over the past three decades. The focus on changes on the supply-side is clearly motivated by Fig. 1 which shows a dramatic increase in the shares of graduates and immigrants since the mid-1990s,^2^ boosted respectively by a large expansion in higher education places in the early 1990s and the EU enlargement of 2004.

Goos and Manning (2007) used data for 1979–1999 and concluded that compositional changes could not explain the polarisation in the UK. This highly influential paper remains the only paper to have tackled this question [see McIntosh (2013) for a review]. This paper replicates and extends to more recent years the work of Goos and Manning (2007) and provides new insights on the polarisation process in the UK. In particular, the main contribution of this paper is to provide new evidence on the role of changes in the skill mix exploring for the first time the contribution of individual skill groups to the aggregate pattern of polarisation in general and to changes in the share of *routine* employment in particular. This new evidence provides a more nuanced picture of the implications of changes in the composition of labour supply for the occupational structure than in Goos and Manning (2007) and shows whether any particular feature of the polarisation process can be explained by changes in the relative size of particular skill groups.

As widely recognised in both the labour and trade literature, changes in the skill mix of labour supply can lead to changes in the occupational structure as firms switch to production methods making a more intensive use of the more abundant factor. Dustmann and Glitz (2015) and Lewis (2011) exploit exogenous variation in the supply of immigrants to provide causal evidence that firms adjust production methods to changes in the supply of skills, but isolating exogenous variations in the supply of graduates has generally proven more difficult.^3^ Recently, Blundell et al. (2016) argues that firms adjustment of their occupational structure to the largely exogenous increase in the supply of graduates over the same time period considered in this paper has led to stable educational premia in the UK. The literature on job polarisation has long acknowledged the potential role of changes in the skill mix as well. As mentioned above, Goos and Manning (2007) concluded that changes in the composition of the workforce cannot explain job polarisation in the UK between 1979 and 1999.^4^ It is therefore clear from the existing literature that changes in the skill mix of the workforce are a plausible driver of changes in the occupational structure that deserve empirical consideration.

The main evidence presented in this paper on the role of individual skill groups comes from a shift-share analysis which highlights the contribution to overall job polarisation of changes within and between gender-education-age-immigration cells from 1979 to 2009. Goos and Manning (2007) used a version of this approach to show that compositional changes could not explain the overall polarisation of the labour market between 1979 and 1999. They did not consider the role of immigrants (whose share had only increased marginally by 1999) and their results might not capture fully the cumulative effect of the strong growth in graduates that started a few years into the 1990s (Fig. 1). In addition, due to the data limitations of the time, Goos and Manning (2007) did not consider changes in routine employment within the UK at all.^5^ I use several alternative measures of routiness (Autor 2013) to provide the first evidence on the extent to which compositional changes can account for the decline in *routine* employment specifically.

Importantly, changes in the relative size of different groups could account for significant features of the polarisation process even if they do not explain the entire pattern as observed by Goos and Manning (2007). To investigate this hypothesis, I present a breakdown of the shift-share analysis which provides the first evidence for the UK on the contribution of different skill groups to changes in employment shares across the occupational skill distribution in each of the past three decades.

The evidence on the contribution of different skill groups to polarisation is of interest for at least two reasons. First, in the absence of credible sources of exogenous variation on either side of the labour market, assessing the relative importance of within vs between group polarisation is crucial for a critical appraisal of the hypothesis that the process is driven by technology. This is why Goos and Manning (2007) use this method, and, more generally, evidence of pervasive polarisation within different skill groups is typically cited in support of a technology effect as it indicates that aggregate polarisation is not merely the result of changes in the relative size of groups specialising in different occupations (Spitz-Oener 2006; Acemoglu and Autor 2011). Second, the evidence on the contribution of individual groups to the aggregate polarisation enhances our understanding of the specific challenges faced by different skill groups in an increasingly polarising labour market.^6^ Evidence on the contribution of individual skill groups to aggregate polarisation is currently very limited for countries other than the US as I discuss in Sect. 2.

The key findings of the paper can be summarised as follows. The main feature of the polarisation process in the UK has been a shift of employment towards high-paid occupations, which have gained 80% of the employment shares lost by middling occupations. When occupations are ranked by education, it becomes apparent that it is those with the lowest initial level of education that have lost most employment shares. The results of the shift-share analysis suggest that the increase in the share of graduates has contributed significantly to this substantial reallocation of employment from middle-pay to high-pay occupations. The increase in immigrants, on the other hand, does not explain any particular feature of job polarisation in the UK. In addition, there is no clear indication of polarisation *within* all skill groups—a fact that previous literature has cited as evidence supporting the hypothesis that technology is the main driver of the process. Hence, this paper adds to the growing body of evidence from other countries (Autor 2015; Green and Sand 2015) that casts doubts on the extent to which technology can be seen as the primary driver of the polarisation process.

## Related literature

Goos and Manning (2007) were the first to propose a link between job polarisation and technological change. They argued that the hollowing out of the UK labour market is due to the concentration in middling occupations of “routine” tasks which are easier to automate because they can be executed following a precise set of standard instructions (Autor et al. 2003).

Later studies documented job polarisation in the US (Autor et al. 2006), Canada (Green and Sand 2015), Sweden (Adermon and Gustavsson 2015), Germany (Antonczyk et al. 2018; Kampelmann and Rycx 2011) and across Western Europe (Goos et al. 2009) and pointed to increasing global trade as a possible concurrent cause of the process. Studies that have compared the explanatory power of technology vs offshoring in explaining the decline of routine employment have generally concluded that the latter is more important than the former (Acemoglu and Autor 2011; Goos et al. 2014; Akçomak et al. 2016).^7^

Demand-based explanations for job polarisation imply that middling occupations should experience a decline in both employment shares and wages as a result of a fall in demand, generating both job and wage polarisation. This pattern, however, is only found in the US in the 1990s (Autor et al. 2006; Acemoglu and Autor 2011), but there is no evidence of the contemporaneous occurrence of job and wage polarisation in the 2000s in the US nor at any other time in any of the countries studied in the literature (Dustmann et al. 2009; Mishel et al. 2013; Autor 2015; Green and Sand 2015).^8^ For the UK in particular, the working paper version of this article provides evidence that occupational wage growth has not polarised over time (Salvatori 2015).^9^

Autor (2015) provides an in-depth discussion of this and other puzzles confronting the routine-biased technological change hypothesis (and demand-based explanations more broadly), but here suffice it to say that the heterogeneity of employment and wage patterns across countries and over time suggests that factors other than technology continue to play a significant role. For instance, Autor (2015) and Green and Sand (2015) have emphasised that recent developments in occupational wages at the bottom of the distribution (in the US and Canada respectively) appear consistent with significant shifts in labour supply as well.

More generally, the potential role of changes on the supply side of the labour market have long been acknowledged in this literature in light of the previous evidence from the trade and labour literature that firms adjust their production technologies to changes in the skill mix of the workforce.^10^ As discussed in the Introduction, Goos and Manning (2007) used a shift-share analysis to address this issue in their study on the UK for the years 1979–1999. Oesch (2013) offers an analysis similar to that of Goos and Manning (2007) for five European countries (including the UK) over the period 1993–2008 which shows that changes in the education and age mix of the workforce are associated with growth in high-skilled occupations. Neither of these studies includes immigrants in the compositional analysis, although Oesch (2013) documents the increase in their numbers and acknowledges their potential role.

As noted by Autor and Dorn (2009), job polarisation might be affecting different skill groups in different ways. Most (if not all) of the direct evidence on this issue, however, comes from the US where the decline in middle skill employment of non-college workers has mostly been compensated by increases in bottom occupations, while for college workers both low skill and high skill occupations have grown (Autor and Dorn 2009). No comparable evidence is reported in existing studies on Germany (Antonczyk et al. 2018; Kampelmann and Rycx 2011), Sweden (Adermon and Gustavsson 2015) and Europe at large (Goos et al. 2009, 2014). For the UK, in particular, both Goos and Manning (2007) and Oesch (2013) focus on aggregate results providing no evidence on the changing occupational structure within individual skill groups or on the contribution of the individual groups to overall polarisation.

## Data and occupational coding

I use four different datasets covering the period 1979–2012. Data on occupational shares and socio-demographic characteristics come from the Labour Force Survey (LFS) which was carried out biannually from 1979 to 1983, then annually from 1984 to 1991, and finally quarterly from 1992 onwards. Between 1979 and 2012, the LFS uses four different occupational classifications (KOS, SOC90, SOC00, SOC10) (ONS 2002). There is some evidence from the US that different ways of bridging occupational classifications might lead to different results (Lefter and Sand 2011) but the issue has not been investigated with UK data. I use probabilistic matching and investigate the sensitivity of the conversion procedure to conditioning on different observable characteristics. To the extent that groups grow over time at the different rates, different ways of reallocating them across occupations might affect the estimates of changes in the size of occupations over time. Section 4 shows the results obtained converting across occupational classifications conditionally on education and unconditionally (as in Goos and Manning 2007) and the interested reader is referred to Additional file 1: Appendix S1 for more details. In addition, Additional file 1: Appendix S2 discusses the issues encountered in the LFS when measuring the share of graduates over time and the education of foreign-born workers. To maximise comparability with existing studies and facilitate the application of the shift-share analysis, I follow Goos and Manning (2007) and focus on changes in occupational shares by employment deciles at the beginning of the observation period.^11^

Because the LFS did not collect data on earnings until 1993, the wage data come from the panel dataset combining the New Earnings Survey (NES, 1979–2002) and its successor the Annual Survey of Hours and Earnings (ASHE), available from the UK Data Archive as NESPD. Wage information is provided by the employer and is therefore regarded as very reliable, but this dataset has no education variables and very limited information on workers’ characteristics in general.

## Employment polarisation over time in the UK

Building on Goos and Manning (2007), this section documents the evolution of polarisation in the UK over the past three decades and offers new and important robustness checks that have been carried out for other countries but not for the UK. In particular, as discussed in Sect. 3, it considers the robustness of the results to different ways of bridging breaks in the occupational classifications and to ranking occupations by education rather than wages.

**Fig. 2:**
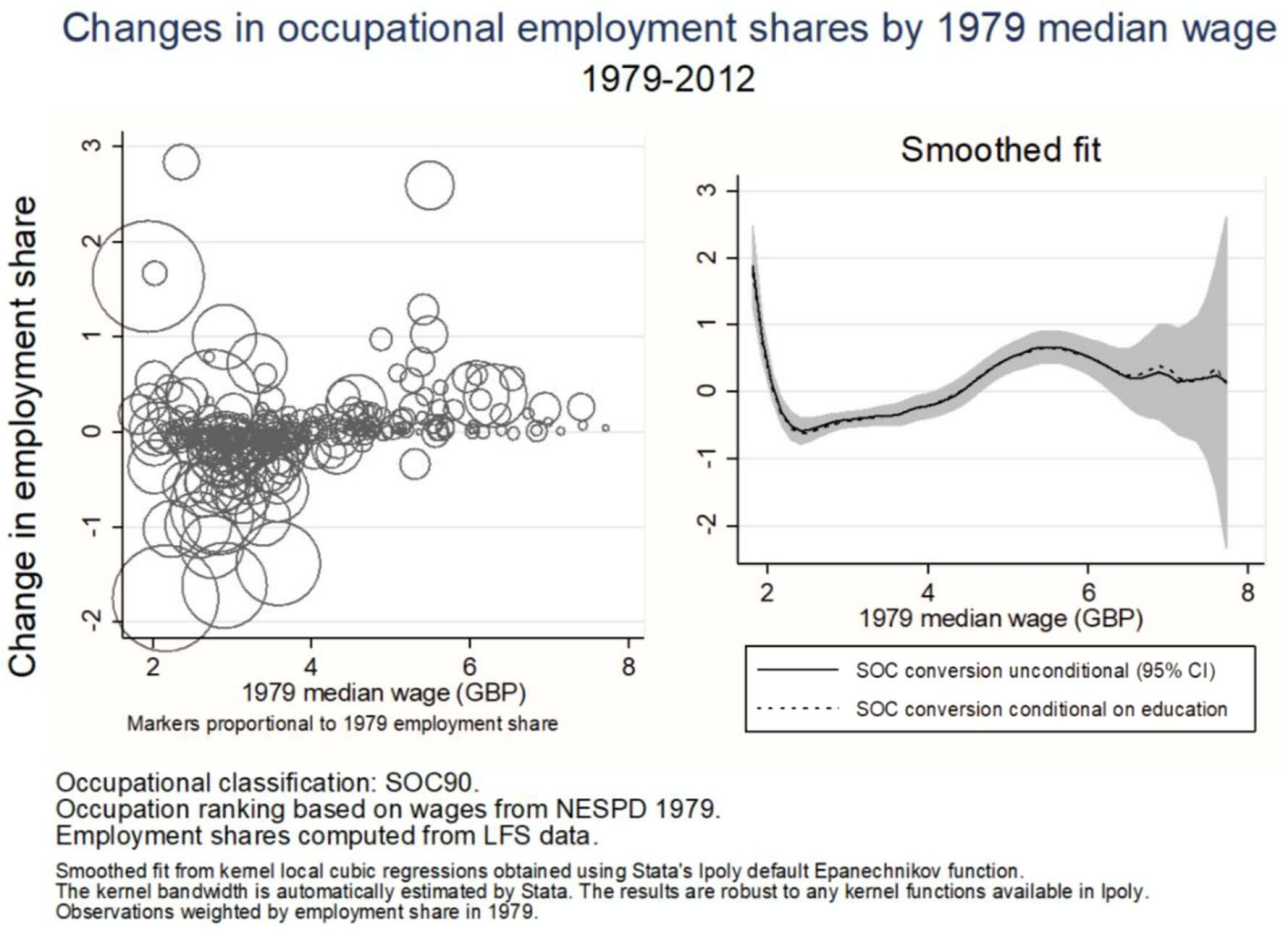
Changes in occupational shares by 1979 median wage, 1979–2012

Figure 2 shows the changes in employment shares across occupations along the 1979 occupational wage distribution. The figure on the left shows the percentage point change in employment share for each occupation, with markers proportional to the occupational employment share in 1979. The figure on the right shows a smooth line fitted non-parametrically through the points on the left, with each observation weighted by employment share in 1979. The solid black line is the fit for the employment changes obtained when the occupational conversion is done unconditionally, while the dotted line is the fit for the employment changes obtained with the conversion conditional on education. The two lines are very similar, except for the fact that, as expected, the conversion conditional on education shows a slightly higher growth for some of the top-paid occupations. On the whole, however, these differences do not affect the general conclusion: employment growth exhibits a polarised pattern between 1979 and 2012. In the remainder of the paper, I present only results obtained using the unconditional conversion method. More generally, the polarisation result survives a number of robustness checks, including (i) ranking occupations by mean rather than median wages, (ii) using hours share rather than employment shares, (iii) using different base years for the occupational rankings, and (iv) occupational classifications other than SOC90.

**Fig. 3:**
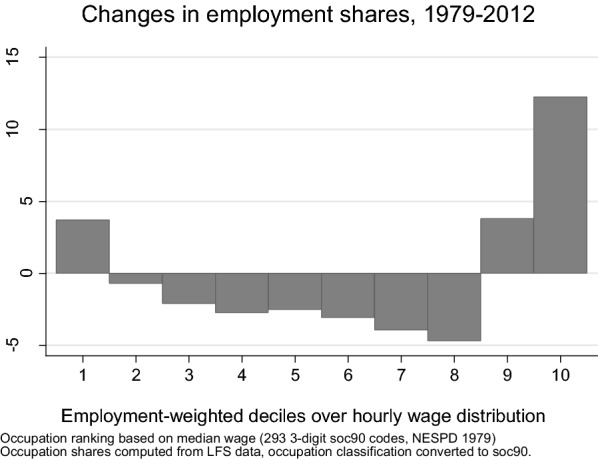
Changes in employment shares by occupational deciles, 1979–2012

Following Goos and Manning (2007), in Fig. 3 occupations are grouped into employment-weighted deciles of the 1979 wage distribution. Only the occupational deciles at the two extremes of the distribution gained shares between 1979 and 2012, with an overall shift of employment mostly directed towards high-skill occupations. The largest growth occurred in the 10th decile which more-than-doubled its relative size over this period. Overall, of the 19 pp of employment share lost in middling occupations, 16 pp have been gained by top occupations and only 3 by bottom ones.

**Fig. 4:**
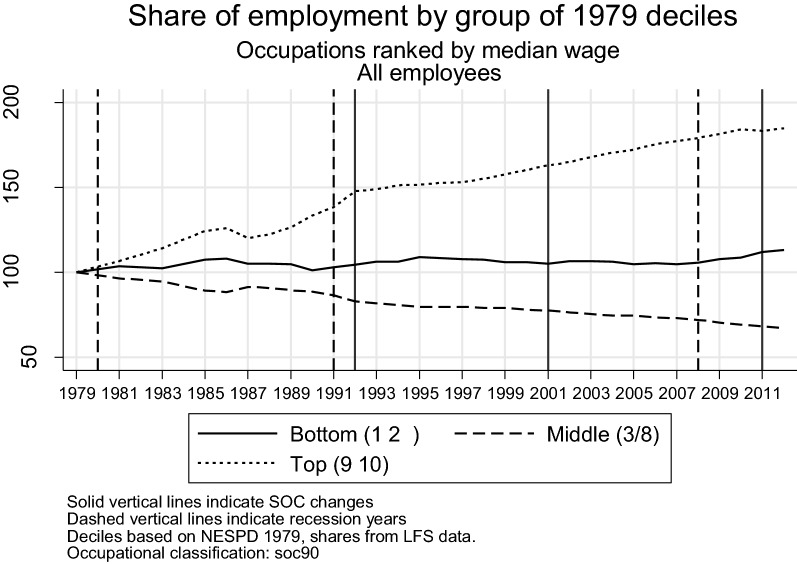
Occupational shares by group of 1979 deciles

Figure 4 plots the time series of the employment shares of the bottom (1st and 2nd deciles), middling (3rd to 8th deciles) and top (9th and 10th deciles) occupations normalised by their starting value in 1979. The dashed vertical lines indicate recession years, while the solid lines mark changes in occupational classifications in the LFS data. The UK experienced job polarisation in each of the last three decades, as middling occupations declined in a relatively steady manner between 1979 and 2012, losing about a third of their initial share. Job polarisation appears to be a long-term process not heavily affected by the business cycle.^12^ Bottom occupations fluctuated above their initial share for most of the period and had grown by 10% by 2012. The share of top occupations grew by more than 80%, with more than half of that growth occurring in the first 13 years covered by the data.^13^

### Changes in employment shares along the educational distribution

**Fig. 5:**
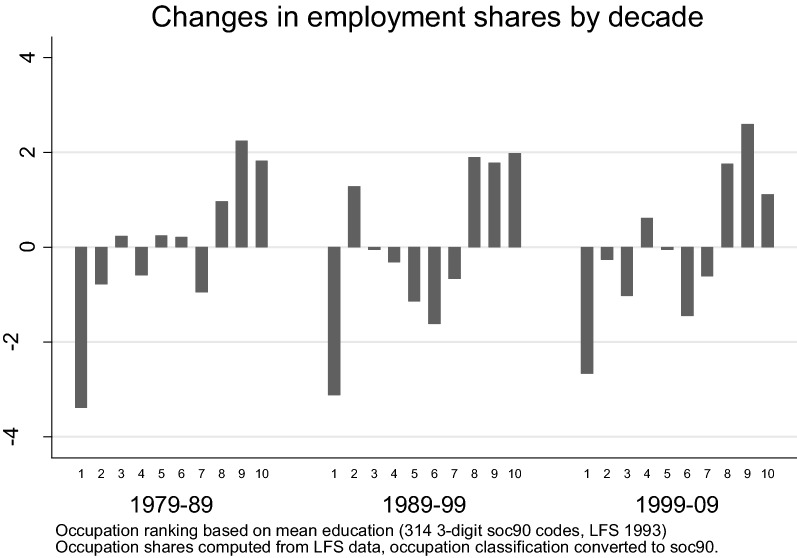
Decadal changes in employment shares by deciles of the 1993 education distribution

The robustness of the polarisation result to the use of education rankings rather than wage ones has been verified for the US and Canada (Acemoglu and Autor 2011; Green and Sand 2015), but not for the UK. This is an important check because wages are typically used as proxies for skills in this framework. Figure 5 shows that when occupations are ranked by mean education rather than wage, the occupations in the first decile are the ones losing the largest shares and those at the top make the largest gains. Figure 5 uses the education ranking from the year 1993 because that is the first year with the original SOC90 classification in the LFS. Additional file 1: Appendix S2 shows that *wage* rankings from 1993 return the usual polarised pattern and documents the differences between the wage and education rankings in 1993.

The difference in the results by wage and education rankings is in contrast with previous evidence for the US (Autor et al. 2006) and Canada (Green and Sand 2015). While the wider implications of the differences between the results by wage and education warrant further research, for the purpose of this paper I stress that the presence of a clear upgrading patter in occupations by education provides the first indication that increasing educational attainment might have contributed to shaping the changes in the occupational structure of the UK. The next section presents the results of a shift-share analysis to shed more light on this issue.

## The contribution of compositional changes to employment polarisation: a shift share analysis

This section presents the main results of the shift-share analysis used to investigate the extent to which the changes in the composition of the workforce highlighted in Fig. 1 have contributed to job polarisation in the UK. As discussed in the Introduction, this method is very closely related to that used in Goos and Manning (2007) to argue that compositional changes do not explain the broad polarisation pattern. Here, the analysis is extended to include immigrants and the results are broken-down by different skill groups to highlight the contribution of each of them to different aspects of the overall polarisation pattern. This yield a new and more nuanced picture of the potential role of compositional changes than in Goos and Manning (2007), one that is also informative of the challenges and opportunities facing different skill groups.

The shift-share analysis decomposes changes in the employment share of decile *o* between *t*_0_ and *t*_1_ (Δ*S*_*ot*_) as follows:

1 $$\Delta S_{ot} = \varSigma_{\text{g}} \Delta S_{gt} \omega_{ogt} + \varSigma_{\text{g}} \Delta \omega_{ogt} S_{gt}$$

$\omega_{ogt }$ is the average share of group *g* employed in decile *o* between *t*_0_ and *t*_1_ and *S*_*gt*_ is the average employment share of demographic group *g* over the same period. Δ is the difference operator between *t*_0_ and *t*_1_. The first term on the right-hand side of Eq. (1) is the between component, i.e. the change in employment share accounted for by changes in the shares of different skill groups (or compositional changes), holding constant the distribution of each skill group across the occupational deciles. The second term is the within-group component. i.e. the change in occupational share due to changes in the distribution of the skill groups across occupations, holding constant the relative size of the skill groups. Importantly, this latter term allows us to see whether polarisation has occurred within different skill groups.

### Aggregate results

**Fig. 6:**
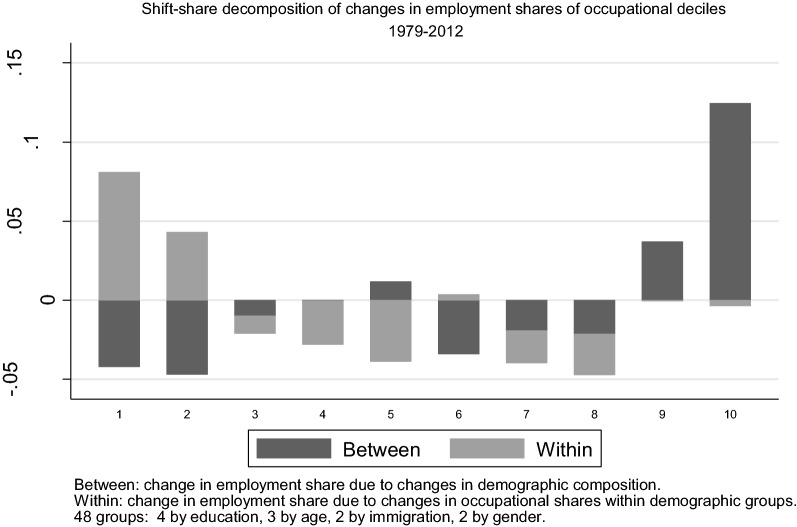
Shift-share decomposition of changes in employment shares of 1979 occupational deciles

Figure 6 reports the results of the shift-share analysis using 48 skill groups defined by education-age-immigration-gender cells. In particular, I use 3 age groups (under 30, 31–50, and over 50) and 4 education groups: university or higher education qualification (for convenience I refer to this group as “*graduates*”); GCE (this the secondary education qualification required of students wanting to access University education in the UK); GCSE and other qualifications; and no qualifications.

A clear message from the picture is that the changes in the composition of the workforce have led to a reallocation of employment shares towards top occupations, while changes in the allocation of skill groups across occupations have fuelled the relative growth of the bottom. It is the combination of these two forces that has led to the overall polarisation of the labour market between 1979 and 2012.

Table 1 shows that the relative size of the between and within-group components remains stable when using just 4 education groups or as many as 400 skill cells. For the remainder of the paper I focus on the results based on the 48 groups described above.

**Table 1:** Shift-share decomposition of changes in occupational shares (pp) by different set of groups

	Total^d^	Education only		Education, age, gender, immigration		Education, age, gender, immigration, geography	
		4 groups^a^		48 groups^b^		400 groups^c^	
		Between	Within	Between	Within	Between	Within
1979–2012							
Bottom	3.5	− 10.2	13.8	− 8.9	12.4	− 8.9	12.4
Middle	− 19.3	− 6.2	− 13.1	− 7.2	− 12.0	− 7.5	− 11.8
Top	15.7	16.4	− 0.7	16.2	− 0.4	16.3	− 0.6
1979–1989							
Bottom	0.9	− 3.6	4.4	− 2.2	3.1	− 2.3	3.1
Middle	− 5.9	− 0.3	− 5.6	− 1.3	− 4.5	− 1.5	− 4.4
Top	5.0	3.9	1.1	3.6	1.4	3.8	1.2
1989–1999							
Bottom	1.0	− 4.2	5.3	− 4.2	5.2	− 4.5	5.5
Middle	− 6.6	− 1.9	− 4.7	− 2.4	− 4.2	− 2.2	− 4.4
Top	5.6	6.1	− 0.6	6.6	− 1.1	6.7	− 1.2
1999–2009							
Bottom	0.3	− 2.8	3.2	− 3.0	3.3	− 2.5	2.9
Middle	− 4.9	− 1.5	− 3.3	− 1.4	− 3.5	− 1.5	− 3.4
Top	4.5	4.4	0.2	4.3	0.2	4.0	0.5

^a^4 education groups (higher + further education, A-level, O-level + other, none)

^b^4 education groups, 3 age groups (< 30, 31–50, > 50), 2 genders, 2 immigrant status

^c^4 education groups, 5 age groups (< 25, 26–35, 36–45, 46–55, > 55), 2 genders, 2 immigrant status, 5 geographies (North, Midlands and EastAnglia, London, South, Scotland + Wales + Northern Ireland)

^d^The between and the within components do not always sum up to the totals due to rounding

As shown in the top panel of Table 1, occupations in the top two deciles grew by almost 16 percentage points (pp) and this is entirely accounted for by compositional changes. The 19 pp decline in middling occupations, on the other hand, is the result of two forces: compositional changes account for about a third of it (about 7 pp) while the shift of employment towards other occupations for the remaining two-thirds (12 pp). This reshuffling of skill groups across occupations has entirely been directed towards the bottom (as clearly visualised in Fig. 6), and has only partially been offset by between-group changes, resulting in a net increase of 3.5 pp at the bottom. The lower panels of Table 1 show that that the pattern of between and within skill group changes is similar in each of the 3 decades since 1979.

The next section presents the results of the shift-share analysis focusing on the two groups whose relative size has changed most dramatically in recent times, i.e. graduates and immigrants (Fig. 1). Results by age groups and gender can be found in the Additional file 1: Appendix S3.

### Results by education and immigration status

The breakdown by graduates vs. non-graduates in columns 4 through 9 of Table 2 shows that the entire decline in middling occupations is accounted for by non-graduates. They contributed − 28 pp to the change in the employment share of middling occupations, over half of which is explained by the decline in their relative number (15.5 pp). The compositional change has been the driving force in the decline of non-graduate employment since the 1990s, as indicated in the lower panels in Table 2.

**Table 2:** Contributions of skill groups to changes in employment shares (pp) across the occupational distribution

	All			Graduates			Non-graduates			Natives			Immigrants		
	(1)	(2)	(3)	(4)	(5)	(6)	(7)	(8)	(9)	(10)	(11)	(12)	(13)	(14)	(15)
	Total	Between	Within	Total	Between	Within	Total	Between	Within	Total	Between	Within	Total	Between	Within
1979–2012															
Bottom	3.5	− 8.9	12.4	3.9	3.1	0.8	− 0.4	− 12.0	11.6	0.6	− 10.1	10.7	2.9	1.2	1.7
Middle	− 19.3	− 7.2	− 12.0	9.0	8.3	0.7	− 28.3	− 15.5	− 12.7	− 20.6	− 10.3	− 10.4	1.4	3.0	− 1.7
Top	15.8	16.2	− 0.4	15.0	16.6	− 1.5	0.7	− 0.4	1.2	11.9	12.3	− 0.4	3.8	3.9	0.0
1979–1989															
Bottom	0.9	− 2.2	3.1	0.3	0.4	0.0	0.5	− 2.6	3.1	0.6	− 2.2	2.8	0.3	0.0	0.3
Middle	− 5.9	− 1.3	− 4.5	1.2	1.1	0.1	− 7.1	− 2.4	− 4.6	− 5.5	− 1.5	− 4.0	− 0.4	0.1	− 0.6
Top	5.0	3.6	1.4	2.4	2.5	− 0.1	2.6	1.1	1.5	4.4	3.2	1.2	0.6	0.4	0.3
1989–1999															
Bottom	1.0	− 4.2	5.2	0.3	0.7	− 0.4	0.7	− 4.9	5.6	0.9	− 4.1	5.0	0.1	− 0.1	0.3
Middle	− 6.6	− 2.4	− 4.2	3.6	2.9	0.7	− 10.2	− 5.4	− 4.8	− 6.4	− 2.7	− 3.7	− 0.2	0.3	− 0.5
Top	5.6	6.6	− 1.1	6.2	6.4	− 0.3	− 0.6	0.2	− 0.8	4.6	6.0	− 1.3	0.9	0.7	0.2
1999–2009															
Bottom	0.3	− 3.0	3.3	1.8	0.7	1.1	− 1.5	− 3.7	2.2	− 1.6	− 4.2	2.6	1.9	1.2	0.7
Middle	− 4.9	− 1.4	− 3.5	2.6	2.7	− 0.2	− 7.5	− 4.1	− 3.3	− 6.6	− 3.2	− 3.3	1.7	1.8	− 0.2
Top	4.6	4.3	0.2	4.5	5.4	− 0.9	0.1	− 1.0	1.1	3.0	2.3	0.7	1.6	2.1	− 0.5

Results from a shift-share analysis

The table reports the total by education groups from the shift-share analysis with 48 skill groups. Immigrants are defined as foreign-born workers

*Within* the non-graduate group, employment has polarised but with a major shift from middling to bottom occupations. Between 1979 and 2012, this shift has contributed to the loss of 12.7 pp (column 9) in middling occupations which have been mostly reallocated to bottom occupations (11.6 pp). Hence, in 2012 there were fewer non-graduates (relative to graduates) than in 1979 and they were much more concentrated in bottom occupations.

Graduates, on the other hand, have made monotonically increasing positive contributions to the growth of all three segments of the occupational distribution (column 4). The shift in graduate employment away from top occupations (column 6) is very small compared to the impact on aggregate employment of the large expansion in graduate numbers (column 5). For example, between 1979 and 2012 the increase in the share of graduates accounted for a 16.6 pp growth in top occupations, while their reallocation towards lower occupations only subtracted 1.5 pp. The within-group change among graduates has been negative in each decade at the top, while in the middle it is only negative (− 0.2 pp) in the most recent decade. As a result, the 2000s is the decade in which the shift of graduate employment towards the bottom is most pronounced. Overall, therefore, there is no polarisation *within* graduates (column 6)—a notable fact since the occurrence of polarization within all skill groups is often cited as evidence consistent with a technology effect (Spitz-Oener 2006; Acemoglu and Autor 2011).

The first row of Table 2 shows that graduates account for the growth in bottom occupations between 1979 and 2012. Although a higher proportion of non-graduates is found in bottom occupations in 2012 than in 1979, the reduction in the overall number of non-graduates means that overall fewer of them are found in these occupations as a fraction of all employees, as indicated by their net overall contribution of − 0.4 pp (column 7). Graduates have more-than-offset this decline through both between- and within-group changes. The lower panels of the table show that the contribution of graduates to the growth of bottom occupations only exceeded that of non-graduates in the 2000s.

The breakdown by immigration status (column 10 through 15) shows that the role played by foreign-born workers has increased over time. Most of the long-term results reported in the top panel of the table are driven by the 2000s. Immigrants contributed to the growth of all three segments of the occupational distribution, with larger impacts at the two extremes hence sustaining the overall polarisation of the occupational distribution.

Over 30 years, the distribution of employment among immigrants has shifted towards the bottom (column 15). This is the result of a polarised pattern in the 1980s and 1990s, followed by a more pronounced downgrading one in the 2000s, as foreign-born workers lost shares at the top as well as in the middle.

The increase in the number of immigrants between 1979 and 2012 does not account for any particular feature of the polarisation process per se. The increased number of foreign workers contributed to the growth of all occupational groups, but more so in top and middling occupations than bottom ones (column 14). This leads to the result that immigrants’ largest contribution in absolute terms has occurred at the top, where they account for 3.8 pp of the total 15.8 pp increase in employment share (column 13).

However, it is at the bottom that they made the largest contribution relative to natives, as immigrants account for 4/5 of the 3.5 pp net increase in employment shares in these low-pay occupations—a result driven by the 2000s.

In Table S4 (Additional file 1: Appendix S3), I present a further breakdown of the results by education-immigration cells that clarifies two points. First, the growth at the bottom is not accounted for by the fact that highly educated foreigners end up in low pay occupations, as immigrants account for only 1.2 pp of the 3.9 pp graduate contribution to the growth in these occupations. Second, by far the largest positive contribution to growth of employment in low-pay occupations has come from the reallocation of native non-graduates from middling to bottom occupations. In fact, this latter change alone almost entirely offsets the negative compositional change arising from their decline relative to graduates.

Things are different in the 2000s, when the share of natives employed in low-pay occupations declined for the first time and bottom occupations only grew due to the contribution of immigrants (columns 10 and 13, bottom panel, Table 2). Nevertheless, even in the most recent decade growth at the bottom is not explained by compositional changes only. The net contribution of improvements in education and increase in immigration is a negative 3.0 pp (column 2, bottom panel, Table 2). This is more-than-offset by the positive 3.3 pp change stemming from the fact that all groups have increasingly been drawn to the bottom (column 3). What distinguishes the 2000s from the two earlier decades is that this reallocation of workers from the middle to the bottom affects graduates as well, as the comparison of columns 6 and 9 across the panels of Table 2 shows. Additional file 1: Appendix S3 further clarifies that it is both native and immigrant graduates who have seen their employment shift towards the bottom in the last decade.

In conclusion, our detailed shift-share analysis returns a number of important and novel results that can be summarised as follows: the improvements in educational attainment have sustained the shift from middling to top occupations, while the reallocation of workers across occupations within skill groups has led to a substantial shift from the middle to the bottom. Polarisation has occurred within non-graduates (although heavily skewed towards the bottom), but not within the graduate group. The decline in middling occupations is entirely accounted for by non-graduates who have both decreased in numbers and seen their employment become more concentrated at the bottom. In the 2000s the share of native workers employed in low-pay occupations declined for the first time and graduates (both natives and immigrants) also saw their employment shift towards the bottom.

## Routine occupations vs non-routine occupations

According to the RBTC hypothesis, the decline in middling occupations is due to the concentration in those jobs of routine tasks which are easier to automate. In fact, this notion has been so widely accepted that measures of routiness are often used as proxy for technology itself (Goos et al. 2014) and changes in routine employment are interpreted as driven by technology. In this section I investigate the extent to which changes in routine employment can be accounted for by compositional changes. I use different classifications of routine occupations based on all three main approaches described in Autor (2013) since no single classification is clearly superior to the others. Here, I offer a brief overview of these three approaches while the interested reader is referred to Additional file 1: Appendix S4 for a more in-depth discussion.

The first approach simply uses occupations as proxies for job tasks, classifying 1-digit occupations based on the task perceived as typical of that occupation. Following Acemoglu and Autor (2011), I classify as routine the following groups: (i) clerical and secretarial occupations, (ii) craft and related occupations, (iii) sales occupations and (iv) plant and machine operatives.

The second approach measures the relative importance of different task dimensions (e.g. routine, abstract, manual) using standardised job descriptors that provide information on the tasks performed in each occupation. I use the “routine task index” (RTI) constructed by David and Dorn (2013) using the 1977 Dictionary of Occupational Titles (DOT) and then mapped to the European ISCO88 (2-digit) classification in Goos et al. (2014, Table 1, henceforth GMS).

The third approach uses job task information collected directly in the British Skill Survey (BSS) to construct an RTI index in the same fashion as David and Dorn (2013) as described in Additional file 1: Appendix S4. To minimise the role played by my own subjective judgement in classifying the 36 available tasks as either routine or non-routine (Green 2012), I follow Akçomak et al. (2016) (henceforth AKR) who use a subset of the tasks split in groups intended to reflect those defined by the task measures in David and Dorn (2013).

Table 3 shows the results of the shift-share analysis for routine and non-routine occupations separately using three alternative classifications: the one based on the Acemoglu and Autor (2011) and the two using the top employment-weighted 30% occupations based on the GMS and AKR RTI indexes.

**Table 3:** Shift-share decomposition of changes in occupational shares (pp) by type of occupations using alternative routine classifications, 1979–2012

	Non routine occupations			Routine occupations		
	Total	Between	Within	Total	Between	Within
(A) Routine classification based on Acemoglu and Autor (2011)						
Bottom	4.2	− 4.6	8.8	− 0.7	− 4.3	3.6
Middle	2.6	1.6	1.0	− 21.8	− 8.8	− 13.0
Top	16.6	16.3	0.3	− 0.8	− 0.1	− 0.7
All				− 23.4	− 13.2	− 10.1
(B) Routine classification based on RTI index from Goos et al. (2014)—top 30%						
Bottom	6.1	− 6.9	12.9	− 1.6	− 1.1	− 0.4
Middle	− 7.9	− 2.9	− 4.9	− 11.5	− 1.4	− 10.1
Top	14.9	12.3	2.6	0.0	0.0	0.0
All				− 12.7	− 3.4	− 9.3
(C) Routine classification based on RTI index from Akçomak et al. (2016)—top 30%^a^						
Bottom	6.5	− 4.2	10.7	− 3.0	− 4.7	1.7
Middle	− 6.7	− 1.5	− 5.1	− 12.6	− 5.7	− 6.9
Top	15.9	16.2	− 0.3	− 0.1	0.0	− 0.1
All				− 15.7	− 10.5	− 5.3

Results from a shift-share analysis with 48 groups: 4 education groups, 3 age groups, gender, immigration status. Details on the routine classifications are provided in Sect. 6 and Additional file 1: Appendix S4

The discrepancies between the totals in Panel B and the other two panels is due to the fact that the RTI index from Goos et al. (2014) is only available for 21 ISCO 88 codes

^a^Due to the size of the underlying occupations, the actual initial share of routine occupations here is 40% as shown in Table 3

Routine occupations have declined relative to non-routine under all classifications. The comparison with the 1979 totals reported at the bottom of Table 3 indicates that the fall in routine-occupations is substantial as they have lost around 40% of their employment shares across classifications.^14^ In panels A and C, most of the decline in routine occupations is accounted for by compositional changes. In Panel B, within-group changes are more important, but the result is not robust to the use of the alternative classifications based on the same GMS RTI index. In all cases, routine occupations account for most of the decline in middling occupations and their contribution here is mostly from within-group changes.

Overall, therefore, two main conclusions can be drawn. First, routine occupations have declined relative to non-routine occupations and they account for most of the decline in middling occupations. Second, the overall decline in routine occupations is mostly accounted for by between-group changes, but the contribution to the decline in middling occupations is instead driven by within-group changes.

## Summary and conclusions

As discussed in Sect. 2, the US is the country on which most of the existing literature on job polarisation has focused. As it is customary in related papers (Green and Sand 2015, Antonczyk et al. 2018), it is therefore useful to refer to the US results when discussing those of other countries. This paper shows that the evolution of job polarisation in the UK appears substantially different from that documented for the US in previous literature. While employment growth in the US has progressively favoured bottom occupations and was only polarised in the 1990s, in the UK polarisation occurred in each of the last three decades and growth in high-skill occupations has always exceeded that in low skill ones. Overall, between 1979 and 2012, top occupations gained about 16 of the 19 pp of employment shares lost by middling occupations.

What exactly drives the patterns observed in the US is the subject of an on-going debate (Autor 2015; Beaudry et al. 2016) and beyond the scope of this paper, but the differences between two similarly developed countries suggest that factors other than (broadly similar) technological change might be at play (Green and Sand 2015; Antonczyk et al. 2018). The results of the shift-share analysis indicate that the increase in the educational attainment of the workforce is likely to have contributed significantly to the most prominent feature of the polarisation process in the UK, i.e. the substantial reshuffling of employment from middling to top occupations.

Unlike in the US (Autor and Dorn 2009; Autor 2014), graduates have played no role in the decline of middling occupations, but the increase in their numbers accounts for the entire growth in the top ones from the 1990s—when their growth accelerated dramatically. There is some polarisation within non-graduates (but heavily skewed towards the bottom), but no polarisation within graduates. This is a notable fact since pervasive polarisation within skill groups is cited in the literature as evidence of a technology effect (Spitz-Oener 2006; Acemoglu and Autor 2011). Overall, changes in the relative size of skill groups account for about a third of the decline in middling occupations and for most of the decline in *routine* occupations across the whole distribution. In addition, I find that the occupations that have lost the largest employment shares are those with the lowest initial level of education. This is a result in stark contrast with that for the US (Autor et al. 2006) and again points to the likely importance of changes in the education mix of the workforce. Moreover, the working paper version of this article (Salvatori 2015) finds no evidence of the polarisation of the occupational wage growth that has been interpreted as evidence in favour of the RBTC hypothesis in previous literature (Autor et al. 2006; David and Dorn 2013). This is in line with results from other countries such as Canada (Green and Sand 2015) and Germany (Dustmann et al. 2009; Antonczyk et al. 2018; Kampelmann and Rycx 2011).

These results indicate that the increase in the share of graduates might help explain why the UK continued to see growth in top occupations in the 2000s when the evidence from the US suggests that technology-driven demand for cognitive skills slowed down (Beaudry et al. 2016). I also find that in the last decade the employment structure of graduates in the UK shifted towards the bottom, a result consistent with the hypothesis that the supply of high skills might have outgrown the demand arising in top occupations. Similar results have been found for Germany for the latest decade (Reinhold and Thomsen 2017). In addition, two recent papers focusing on the UK have also provided evidence that the market for graduates in the UK might be near saturation. Green et al. (2016) report on the rising prevalence of over education and Blundell et al. (2016) argue that a decline in the graduate wage premium since 2010 might be indicative that firms’ ability to absorb the increasing supply by adapting their organisational structure might be exhausting.^15^

While the growth of graduates can account for the shift of employment from middling to top occupations, it cannot explain the entire polarisation process. In particular, the employment growth in bottom occupations has occurred *in spite of* the increase in education. Immigrants account for a substantial fraction of *net* growth in these occupations (mostly in the 2000s), but the most significant change offsetting the downward pressure arising from the increase in education is, by far, the reallocation of native workers with intermediate qualifications from middling occupations into service occupations. Wage growth for these occupations was robust over the past 30 years and the highest across all occupational groups in the 2000s (Salvatori 2015).

The presence of both wage and employment growth in bottom occupations is consistent with the explanations that emphasise increased product demand either from high-skill workers or arising from complementarity in consumption between services and the goods made cheaper by technology (David and Dorn 2013; Mazzolari and Ragusa 2013).

The methods employed in this paper are very closely related to those of Goos and Manning (2007) whose conclusion that compositional changes cannot explain the broad pattern of polarisation still informs the understanding of the process in the UK. While this paper also finds that changes in the skill mix alone cannot explain the entire polarisation process, the results indicate that the increase in the share of graduates in the past 30 years has contributed significantly to what is clearly the distinctive feature of the polarisation process in the UK in comparison to the US, namely the substantial reallocation of employment from middling to top occupations. This paper therefore adds to the growing body of empirical evidence from different countries that cast doubts on technology as the dominant driver of polarisation (Autor 2015; Green and Sand 2015).

Admittedly, a convincing causal analysis is impeded by the lack of credible sources of exogenous variations in the supply of graduates that characterises this literature more widely. Changes in the supply of graduates are typically treated as exogenous in related literature (Autor 2014; Card and Lemieux 2001). More specifically, there are strong reasons to doubt that the surge in the share of graduates in the UK was an endogenous response to changes in technology. In fact, this was in large part a stepwise change following the reforms of the late 1980s^16^ which led to a 93% increase in participation in higher education between 1988 and 1996, as opposed to only 15% in the US—arguably the technology-leader (OECD 2007). Blundell et al. (2016) note that the increase in graduates in the UK is unmatched in most developed economies and argue that the puzzling fact that the graduate wage premium has not fallen can be explained by changes in the organisation of work implemented by firms to take advantage of the increasing number of graduates.

In conclusion, while the results of this paper suggest that changes in the skill mix have contributed significantly to re-shaping the occupational structure of the UK in recent decades, a difficult but important task for future research is to overcome data limitations and devise empirical strategies to formally assess the relative contribution of demand and supply factors to the polarisation of the UK labour market.

## Additional file

**Additional file 1:** Appendixes.

## Footnotes

1 For a review and discussion of the puzzles confronting the RBTC hypothesis see (Autor 2015). In Sect. 2, I review some of the most relevant issues for this paper.

2 Additional file 1: Appendix S2 discusses data issues affecting the Labour Force Survey (LFS) in 1992.

3 Beaudry et al. (2010) report evidence that areas with initial higher shares of graduates adopted computers more quickly in the US, and Moretti (2004) uses the lagged city demographic structure and the presence of land-grant colleges to identify the spill-over effects on wages of the supply of graduates across local labour markets in the US.

4 Oesch (2013) offers an analysis similar to that of Goos and Manning (2007) for the period 1993–2008 and considers education-age cells focusing exclusively on the aggregate results for the compositional effects.

5 Instead, Goos and Manning (2007) use US data to show that routine occupations are concentrated in the middle of the occupational distribution in that country.

6 See Sparreboom and Tarvid (2016) for a discussion and analysis of the link between polarisation skill mismatch that also illustrate the importance of understanding whether changing in the occupational structure result from changes in the demand for certain skills by firms or from changes in the supply of such skills.

7 Recent studies have pointed to the importance of international trade more widely. In particular, Autor et al. (2015) and Keller and Utar (2016) have shown that increasing import penetration from China (to the US and Denmark respectively) has contributed to job polarisation by accelerating the decline of manufacturing employment.

8 For Sweden, Adermon and Gustavsson (2015) conclude that the RBTC cannot explain between-occupation changes in wages, but can account for some of the changes within occupations.

9 Goos and Manning (2007) discuss the link between job polarisation and overall wage inequality, but do not report evidence on changes in occupational wages which have been the focus of later papers on other countries.

10 For a theoretical discussion of changes in the supply of different skill groups in the context of a task-based model see Acemoglu and Autor (2011).

11 For a discussion of alternative approaches to measuring polarisation see Sparreboom and Tarvid (2016).

12 In Additional file 1: Appendix S3 I look at the recession periods more closely.

13 I discuss the stark change in the slope of the series in 1992 in Additional file 1: Appendix S2 and conclude data issues likely exaggerate the expansion of top occupations between 1991 and 1992, but the slowdown in their growth in the early 1990 s is likely genuine as also found in other datasets.

14 In the more conservative classifications in panels B and C, some non-routine occupations also appear to have lost shares, but this result generally does not hold when the top employment-weighted 50% occupations by RTI are considered or when all occupations with an RTI above average are identified as routine.

15 For the point about the falling wage premium in recent years see: https://www.ifs.org.uk/uploads/publications/bns/bn185.pdf.

16 See Bolton (2012), Blanden and Machin (2004), Walker and Zhu (2008), Devereux and Fan (2011). More specifically, following a reform of the age 16 examination system in 1988, the share of 17 year old in education climbed from under 30% in 1988 to more than 50% in 1993 greatly expanding the pool of potential university applicants in subsequent years. This change and the expansion in the number of university places available from the early 1990 s also led to a sharp increase in the participation rate in higher education which rose from 19.3% in 1990 to 33% in 2000 (Bolton 2012).
