# Supplementary material for: The anatomy of job polarisation in the UK
Source: J Labour Mark Res. 2018 Jul 25;52(1):8. doi: 10.1186/s12651-018-0242-z (PMC6061237; doi:10.1186/s12651-018-0242-z)
Supplement: Supplementary file 1 — Additional file 1: Appendixes. [file 12651_2018_242_MOESM1_ESM.docx]

Appendix S1 Bridging occupational classifications

Between 1979 and 2012 the LFS used four different occupational classifications. After the Key list of Occupations for Statistical purposes (KOS) between 1979 and 1991, the 1990 Standard Occupational Classification (SOC90) was adopted in 1992, and its successors SOC00 and SOC10 were introduced in 2001 and 2011 respectively. In addition, the NESPD data for 1979-1989 come with a different version of the KOS system. For ease of reference, throughout the paper I will refer to the KOS classification of the early LFS data as SOC80, and to that from the NESPD data as SOC70. Moreover, the occupational classification in which a dataset is originally coded in will be referred to as the “native” occupational classification.

The study of occupational changes over time requires a method to bridge the different occupational classifications. In general, there is no 1-to-1 correspondence even between successive classifications as one (origin) occupation can be split into several (destination) occupations. The ONS (2002) reports that the agreement between any two SOC in dual-coded datasets (i.e. in datasets coded with two versions of the SOC) is always less than 80% even at the 1-digit level.

The availability of a dual-coded dataset offers a way to bridge two classifications by providing estimates of the proportions of a given origin occupation which “move” to different destination occupations. With this information at hand, one can then randomly allocate individuals from a given origin occupation to a set of destination occupations, replicating the empirical distribution observed in the dual-coded dataset^[[1]](#footnote-1)^. This is the method used by Goos and Manning (2007) in their study of polarisation between 1979 and 1999, to convert the occupational coding in the early editions of the LFS (which I refer to as SOC80) to SOC90.

A potential limitation of this approach is that it assumes that the splitting of the origin occupation into the destination occupations is random. Intuition suggests that this is unlikely to be the case since individuals with the same origin occupation are reclassified precisely because they are thought to be doing different jobs and are therefore likely to differ in some respect. For example, it is reasonable that individuals with different skills and education levels will have different probabilities of being assigned to different destination occupations. This can potentially lead to systematic errors in the measurement of occupational shares and their changes if the different demographic groups within an origin occupation grow at different rates. The problem is that the growth of the fastest growing group will be assigned to all the destination occupations in proportions that reflect the demographic composition of the origin occupation in the dual-coded dataset. This will certainly result in an underestimate of the growth in the number of people employed in the destination occupation which actually receive a higher proportion of the fastest growing group, once the non-randomness of the occupational splitting is taken into account. Depending on a number of other factors, including the growth rate of other occupations and demographic groups, this can lead to either over or under estimation of employment shares over time.

For example, suppose that having a university degree is a variable that plays a significant role in the reallocation of individuals between two occupational classifications. It is reasonable that on average graduates within any origin occupation are more likely to be recoded as working in higher-paid destination occupations than non-graduates. As it is well known, the population of graduates in the UK greatly expanded over the past thirty years and in particular in the 1990’s. If one applies an unconditional conversion between the two classifications over this time period, this is likely to result in an underestimate of the share of workers employed in higher-paid occupations since too much of the growth of the graduate population is attributed to lower-paid occupations.

I compared the results using the unconditional conversion method of Goos and Manning (2007) with the three sets of results obtained conditioning the occupational conversion on gender, age and education. I found that the conditional conversion methods do, as expected, effectively reallocate some of the employment growth between occupations – but this takes place among occupations which are close on the wage distribution leaving substantively unaltered the main conclusion that the UK labour market has polarised over the past thirty years. In light of this, the paper only presents the results obtained using the unconditional conversion method.

Appendix S2 Issues concerning the LFS data around 1992 and the measurement of education for immigrants

Assessing the reliability of the LFS time series around the 1992 discontinuity

Over the period 1979-2012, the LFS changes occupational coding three times. The time-series in Figure 4 show that the only point in time where this might matter is 1992, when SOC90 was adopted. However, in the same year the LFS changed in other significant ways. This appendix reflects on the relevance of such changes for the analysis of this paper.

I begin by considering the possible role of breaks in occupational coding in shaping the peculiar pattern in the share of top occupations in the late 1980s and early 1990s. Figure 4 shows that growth in occupations in the highest 2 deciles strongly accelerated in the second half of the 1980s and then abruptly slowed down in the early 1990s. As noted in the paper, this means that more than half of the total increase in top occupations over the 30-year period considered occurred in the first 13 years. The fundamental concern is whether this patter is driven by changes in occupational coding.

To address this issue one would ideally require a dataset with a consistent occupational coding straddling 1992. In the absence of such a dataset, this section compares several different time-series for top occupations. Some of these series are obtained from NESPD/NES data (rather than LFS) and offer consistent occupation coding for at least part of the period of interest, while others are computed using LFS data, but use a different wage rankings which does not require converting across occupational classifications.

As a reminder, the time-series in Figure 4 are all computed using LFS data, with the original occupational classifications from outside the interval 1992-2001 converted to SOC90. Occupations are ranked and split into deciles based on 1979 wages from NESPD data, which are also converted to SOC90.

The first series considered in this section uses a consistent native occupational coding for all the 1980’s. The series is obtained from NESPD data for 1979-1990, using the original occupational coding of that dataset which I label for convenience “SOC70”. The occupational wage rankings are computed from the same dataset in 1979. This is therefore a different occupational classification (and a different occupational wage rankings) from Figure 4.

The second series is obtained from NES data for the period 1990-2001 using the original (unconverted) classification occupation SOC90 for the whole period. The occupational wage ranking is the same as for the series in Figure 4 (1979 NESPD data converted to SOC90). This series straddles the year of the LFS occupation change (1992) maintaining the same original classification, and therefore provides further insights on the behaviour of the series around 1992 net of any conversion issues.

Unfortunately, no dataset offers the possibility of obtaining a wage ranking for the occupations as originally classified in the LFS in the 1980’s^[[2]](#footnote-2)^. It is however possible to rank occupations by the mean education level instead. To avoid further complications, such education rankings are computed for each year only in the original occupational classification of each dataset. This leads to two additional time-series which are useful for the purpose of this section.

The first one comes from LFS data for 1979-1991 and uses the original occupational classification available in those years (KOS, which for convenience I label SOC80) ranking occupations by mean education in 1979. This series therefore uses the same data as the original plot in Figure 4, but with an unconverted occupational classification and a different ranking of occupations.

The second series based on education rankings spans 1979-2001 and uses LFS data in SOC90 (converted from SOC80 for the portion before 1992) and, unlike the other series so far, uses a ranking (based on mean education) from 1993.

Figure S1 plots all of these series, including the original series from Figure 4. The left panel shows the series obtained grouping occupations by the wage ranking (which for all series is based on 1979), while the right panel draws the two series based on education rankings (which in one case refer to 1979 and in the other to 1993).

Net of the relatively small differences that one would expect given the underlying differences between these series, the plots seems to convey a broadly consistent message regarding the behaviour of top occupations in the late 1980’s and early 1990’s.

In particular, in the left panel, we see that both the NESPD and NES series (with their unconverted occupational classification) show steeper slopes in the late 80’s than they do in years immediately before (NESPD) or after (NES). Similarly, the plots in the right panel based on education rankings also show sustained growth in the late 80’s regardless of whether the series is obtained after converting the occupational classification.

Overall, therefore, the graph is reassuring that the behaviour of the time-series for top occupations is not driven by issues related to occupational coding. Most importantly, strong growth in top occupations is seen in the late 1980s using LFS data even when one uses a ranking based on education and the native occupational classification for those years and the data from NES and NESPD (in their respective native occupational classifications) also show a deceleration in the growth of top occupations around 1992.

Nevertheless, Figure S1 makes clear that there is a discontinuity in the LFS series in 1991. Figure 1 shows that in the same year a significant discontinuity is also found in the time-series for the share of graduate employees. In particular, between 1991 and 1992 the share of graduates jumps up by 19% (from 17.6% to 21%), while the average annual growth is around 2% between 1988 and 1991 and around 3% between 1992 and 2012. This discontinuity is not explained by any apparent changes in the coding of the education question in the LFS, nor can be accounted for by changes in the occupational classification. These findings suggest that the changes in the sampling frame of LFS implemented when the survey became quarterly in 1992 might have led to an increase in the estimate of the share of high-skill and high-pay workers.

The dataset documentation mentions three major differences between the pre- and post- 1992 LFS, as the new quarterly survey took on a panel design, introduced an unclustered sample of addresses for the whole of Great Britain, and added people living in NHS accommodation and students in halls of residence to the sample. It is not immediately obvious why any of these changes would generate the discontinuities that we see in the data.

The concern is of course whether these discontinuities are important for the substantive conclusions of the paper. In particular, one might worry that the finding of job polarisation in the 1990s (defined as 1989-1999 in the paper) might be driven by such discontinuities. However, Figure 4 shows that there is still a clear polarisation pattern in employment growth if 1992 is taken as the starting year for the 1990s – in fact there is a slight acceleration in the growth of top occupations and decline of middling occupations in the second half of the 1990s.

Another worry might be that the discontinuity in the series for graduates might affect the conclusions of the shift-share analysis at least for the 1990s (1989-1999) as it might exaggerate the role of between-skill-groups changes. However, the main substantive conclusions on the relative importance of within vs between- group changes across the occupational distribution remains unaltered when the shift-share analysis is carried out for the period 1992-2002 (and 2002-2012).

The education variable for immigrants

A large fraction of university-level qualifications awarded by foreign institutions were coded as “other qualifications” in the LFS until recently. The correction of this issue in 2011 led to an increase of 8-9% foreign-born workers with university education and a corresponding decline in those with “other” qualifications compared to the previous year. This problem likely resulted in underestimate of the growth in the number of graduates in the period of strong immigration growth post 1998 and accounts for the sharp increase in graduates seen in 2011 in Figure 1. However, I found that the main results of the analysis in terms of the relative contributions of different education groups in each decade hold when one consider only the native workers (who are not affected by this misclassification problem)^[[3]](#footnote-3)^. In addition, the results for the long difference between 1979 and 2012 are unlikely to be affected by this issue in a significant way since immigration became quantitatively important only in the late 1990s and the classification problem was rectified in 2011. The paper presents results for the analysis by education groups pooling together natives and immigrants, while a further breakdown by education and immigration status can be found in Appendix S3 .

I did consider the option of defining skill levels using “age left education” as others have done before. However, as acknowledged in the literature, such variable ignores differences in the education systems across countries. In addition, exploratory tabulations revealed a significant degree of variation in “age left education” within groups with the same qualification levels. These two facts indicated that using this alternative classification would likely introduce further noise in the analysis and has led to the decision not to pursue this approach.

Figure S1 - Comparison of different time series for top occupations around the occupation coding break of 1992

Ranking occupations by education

The rankings in Figure 5 are obtained using an average of an education categorical variable taken on 6 values. Education rankings are computed using native workers only. Similar results are obtained when occupations are ranked using the share of graduates, the share with at least high school education and the share with no qualifications. Moreover, the same results are obtained when the rankings (and the change in employment shares) are computed using 2-digit occupations with larger sample sizes. Finally, a clear upgrading pattern for the 1980s is also found when occupations are ranked using 1979 education and the original occupational classification for the LFS in that year. Similarly, if SOC00 and education from 2001 is used, one still sees an upgrading pattern for the 2000s.

Figure S2 shows that *wage* rankings from 1993 return the usual polarised pattern.

**Figure S2 - Decadal changes in employment shares by deciles of the 1993 wage distribution.**

| Table S1 - Contribution of recession periods to changes in occupational shares | | | | | |  |  | |  | |  |  |
| --- | --- | --- | --- | --- | --- | --- | --- | --- | --- | --- | --- | --- |
|  |  | Percentage point change in share of total employment | | | |  | Ratio over 1979-2012 change | | | | | |
|  |  | 1979-2012 | 1979-1983 | 1989-1992 | 2007-2010 |  | 1979-1983 | 1989-1992 | | 2007-2010 | | Total recessions |
|  |  | (a) | (b) | (c) | (d) |  | (e) | (f) | | (g) | | (h) |
|  |  |  |  |  |  |  | (b/a) | (c/a) | | (d/a) | | (e+f+g) |
|  |  |  |  |  |  |  |  |  | |  | |  |
| Bottom (deciles 1-2) | | 3.02 | 0.51 | -0.14 | 0.94 |  | 0.17 | -0.04 | | 0.31 | | 0.44 |
|  | Annualised change | 0.09 | 0.13 | -0.05 | 0.31 |  | 1.40 | -0.49 | | 3.43 | |  |
| Middle (deciles 3-8) | | -19.07 | -3.18 | -3.87 | -2.27 |  | 0.17 | 0.20 | | 0.12 | | 0.49 |
|  | Annualised change | -0.58 | -0.79 | -1.29 | -0.76 |  | 1.38 | 2.23 | | 1.31 | |  |
| Top (deciles 9-10) | | 16.05 | 2.67 | 4.01 | 1.33 |  | 0.17 | 0.25 | | 0.08 | | 0.50 |
|  | Annualised change | 0.49 | 0.67 | 1.34 | 0.44 |  | 1.37 | 2.75 | | 0.91 | |  |

Appendix S3 Further results on polarisation around the recessions and by age and gender groups.

Polarisation and recessions

Table S2 focuses on the contributions of the three UK recessions to the changes in employment shares of different parts of the occupational distribution. Because quarterly data are only available from 1992 onwards I continue to use annual data here. Each of the three recessions lasted four quarters spanning over two calendar years. For the 1990q3-1991q3 and 2008q2-2009q2 contractions, the recession period is defined as the three-year window beginning the calendar year before the start of the recession and ending the calendar year after the end of it (1989-1992 and 2007-2010 respectively for the two recessions). For the 1980q1-1981q1 recession, the longer interval 1979-1983 was taken since LFS data are not available for 1982. It should also be noted that 1979, the initial year of observation, also contains two (non-consecutive) quarters of negative growth (1979q1 and 1979q3) and that the last quarter of 2010 also saw negative growth.

Overall, the three recessions account for about 30% of the period considered and for a higher proportion of the overall change in employment shares (see the last column of Table S2). Half of the 19pp decline in the share of middling occupations and of the 16pp increase in the share of top occupations occurred during recessions. In addition, the annualised decline in middling occupations was larger during each of the three recessions than over the entire 1979-2012 period, with the strongest acceleration occurring in the recession of the early 1990s. However, these estimates are likely to be biased positively by the aforementioned discontinuity in the LFS time-series in 1992 (Appendix S2). In addition, Figure 4 makes clear that the acceleration in the decline of middling occupations (and, even more clearly, in the growth of top occupations) had begun at least in 1988, well before the onset of the recession.

An in-depth analysis of the relationship between polarisation and recessions is beyond the scope of this paper, but these results show that while recessions might have accelerated the polarisation of the labour market, they certainly do not explain the entire process. A similar conclusion is reached by Goos et al (2014) in their online appendix.^[[4]](#footnote-4)^

| Table S2 - Contribution of recession periods to changes in occupational shares | | | | | |  |  | |  | |  |  |
| --- | --- | --- | --- | --- | --- | --- | --- | --- | --- | --- | --- | --- |
|  |  | Percentage point change in share of total employment | | | |  | Ratio over 1979-2012 change | | | | | |
|  |  | 1979-2012 | 1979-1983 | 1989-1992 | 2007-2010 |  | 1979-1983 | 1989-1992 | | 2007-2010 | | Total recessions |
|  |  | (a) | (b) | (c) | (d) |  | (e) | (f) | | (g) | | (h) |
|  |  |  |  |  |  |  | (b/a) | (c/a) | | (d/a) | | (e+f+g) |
|  |  |  |  |  |  |  |  |  | |  | |  |
| Bottom (deciles 1-2) | | 3.02 | 0.51 | -0.14 | 0.94 |  | 0.17 | -0.04 | | 0.31 | | 0.44 |
|  | Annualised change | 0.09 | 0.13 | -0.05 | 0.31 |  | 1.40 | -0.49 | | 3.43 | |  |
| Middle (deciles 3-8) | | -19.07 | -3.18 | -3.87 | -2.27 |  | 0.17 | 0.20 | | 0.12 | | 0.49 |
|  | Annualised change | -0.58 | -0.79 | -1.29 | -0.76 |  | 1.38 | 2.23 | | 1.31 | |  |
| Top (deciles 9-10) | | 16.05 | 2.67 | 4.01 | 1.33 |  | 0.17 | 0.25 | | 0.08 | | 0.50 |
|  | Annualised change | 0.49 | 0.67 | 1.34 | 0.44 |  | 1.37 | 2.75 | | 0.91 | |  |

Shift-share: breakdown by age and gender

Table S3 reports the results of the shift-share analysis by age groups between 1979 and 2012. The increase in the size of older age groups (31-50 and over 50) explains most of the growth at the top. Interestingly, across all age groups the within-group changes appear polarised and skewed towards the bottom. It is however the under-30 who made the largest contribution to the growth of bottom occupations. Although not shown here, this pattern is observed in each of the three decades considered. Over the entire period 1979-2012, the youngest age group accounts for the entire 3.5pp increase at the bottom. This is not the result of mere compositional changes as it is driven by a large within-group change (+7.3pp). In other words, younger people from across all skill groups have increasingly been drawn to the bottom. The under-30s also account for 2/3 of the decline in middling occupations (12pp of the total 19pp) both through between- and within-group changes. The only positive sign found for middling occupations is for the between-group change for the over-50s (+1.1pp). This older group, however, has also seen its employment shift away from middling occupations as indicated by the negative within-group change (-1.6pp), leading to a small negative contribution to the overall change in the share of middling occupations.

The lower panel of Table S3 shows that both genders have contributed to the polarisation of the labour market. Men account for most of the growth in bottom occupations (3.2pp out of 3.5pp), while women for most of the growth at the top (10.2pp out of 15.8pp).

Between-group changes have contributed to the decline at the bottom and the growth at the top for both genders. As for middling occupations, compositional changes have had different effects between the two genders: among men they made a negative contribution, while among women their contribution was positive^[[5]](#footnote-5)^. For both genders the within-group changes have been polarised and for both, on the whole, employment has shifted towards the bottom. In fact, between 1979 and 2012, the within-group contribution to the growth of bottom occupations by the two genders was the same (6.2pp, see Table S3). Men, however, have also moved away from top occupations (-1.7pp) while more women have reached the highest-paying occupations from across the skill groups (+1.3pp). Similar patterns are found in the results by decade, which are not reported here.

| **Table S3 -Contributions of demographic groups to changes in employment shares (pp) across the occupational distribution between 1979 and 2012. Results from a shift-share analysys.** | | | |
| --- | --- | --- | --- |
|  | Total | Between | Within |
| **Bottom** |  |  |  |
| Under 30 | 3.5 | -3.8 | 7.3 |
| 31-50 | -1.0 | -4.4 | 3.4 |
| Over 50 | 1.0 | -0.7 | 1.7 |
| **Middle** |  |  |  |
| Under 30 | -12.1 | -5.7 | -6.5 |
| 31-50 | -6.6 | -2.7 | -3.9 |
| Over 50 | -0.5 | 1.1 | -1.6 |
| **Top** |  |  |  |
| Under 30 | 1.6 | 2.4 | -0.8 |
| 31-50 | 9.6 | 9.0 | 0.5 |
| Over 50 | 4.6 | 4.7 | -0.1 |
|  |  |  |  |
| **Bottom** |  |  |  |
| Men | 3.2 | -3.0 | 6.2 |
| Women | 0.3 | -5.9 | 6.2 |
| **Middle** |  |  |  |
| Men | -15.8 | -11.3 | -4.5 |
| Women | -3.5 | 4.0 | -7.5 |
| **Top** |  |  |  |
| Men | 5.6 | 7.3 | -1.7 |
| Women | 10.2 | 8.9 | 1.3 |
| Totals by demographic groups from the shift-share analysis with 48 skill groups. | | | |

Shift-share: breakdown by education+immigration

| Table S4 - Contributions of skill groups to changes in employment shares (pp) across the occupational distribution. Results broken down by education and immigration status. | | | | | | | | | | | | |
| --- | --- | --- | --- | --- | --- | --- | --- | --- | --- | --- | --- | --- |
|  | **Natives** | | | | | | **Immigrants** | | | | | |
|  | **Graduates** | | | **Non-graduates** | | | **Graduates** | | | **Non-graduates** | | |
| **1979-2012** | Total | Between | Within | Total | Between | Within | Total | Between | Within | Total | Between | Within |
| Bottom | 2.7 | 2.2 | 0.5 | -2.1 | -12.3 | 10.2 | 1.2 | 0.9 | 0.3 | 1.7 | 0.3 | 1.4 |
| Middle | 7.0 | 5.9 | 1.1 | -27.6 | -16.2 | -11.4 | 2.0 | 2.3 | -0.3 | -0.6 | 0.7 | -1.3 |
| Top | 11.5 | 13.1 | -1.6 | 0.4 | -0.8 | 1.2 | 3.5 | 3.4 | 0.1 | 0.3 | 0.4 | -0.1 |
|  |  |  |  |  |  |  |  |  |  |  |  |  |
| **1979-1989** |  |  |  |  |  |  |  |  |  |  |  |  |
| Bottom | 0.3 | 0.3 | 0.0 | 0.3 | -2.5 | 2.8 | 0.0 | 0.1 | 0.0 | 0.2 | -0.1 | 0.3 |
| Middle | 1.1 | 0.9 | 0.1 | -6.5 | -2.4 | -4.1 | 0.1 | 0.2 | -0.1 | -0.5 | 0.0 | -0.5 |
| Top | 2.1 | 2.3 | -0.1 | 2.2 | 0.9 | 1.3 | 0.3 | 0.2 | 0.1 | 0.3 | 0.2 | 0.2 |
|  |  |  |  |  |  |  |  |  |  |  |  |  |
| **1989-1999** |  |  |  |  |  |  |  |  |  |  |  |  |
| Bottom | 0.3 | 0.7 | -0.3 | 0.5 | -4.7 | 5.3 | 0.0 | 0.1 | -0.1 | 0.1 | -0.2 | 0.3 |
| Middle | 3.4 | 2.7 | 0.7 | -9.8 | -5.4 | -4.4 | 0.2 | 0.3 | -0.1 | -0.4 | 0.0 | -0.4 |
| Top | 5.5 | 5.9 | -0.4 | -0.9 | 0.0 | -0.9 | 0.6 | 0.5 | 0.1 | 0.3 | 0.2 | 0.1 |
|  |  |  |  |  |  |  |  |  |  |  |  |  |
| **1999-2009** |  |  |  |  |  |  |  |  |  |  |  |  |
| Bottom | 1.3 | 0.5 | 0.8 | -2.9 | -4.6 | 1.8 | 0.5 | 0.3 | 0.2 | 1.4 | 0.9 | 0.4 |
| Middle | 1.7 | 1.9 | -0.2 | -8.3 | -5.2 | -3.1 | 0.8 | 0.8 | 0.0 | 0.9 | 1.1 | -0.2 |
| Top | 3.2 | 3.8 | -0.6 | -0.2 | -1.5 | 1.3 | 1.3 | 1.6 | -0.3 | 0.3 | 0.5 | -0.2 |
| The table reports the total by education groups from the shift-share analysis with 48 skill groups. | | | | | | |  |  |  |  |  |  |

Appendix S4 Measuring routine occupations

In the spirit of Autor (2013)’s recommendation to use “off the shelf” measures of the routine task intensity of different occupations, I use three different classifications of routine occupations available in the literature.

The first one is based on Acemoglu and Autor (2011) (henceforth AA) and makes use of occupational categories directly rather than resorting to measures at the task level. AA group the US Census and Current Population Survey occupations as follow:

- Non-routine cognitive: managerial, professional and technical occupations.
- Routine cognitive: sales, clerical and administrative support occupations.
- Routine manual tasks: production and operative occupations.
- Non-routine manual tasks: service occupations.

These occupational groups map easily into the 1-digit SOC90 occupational codes. The coarse level of the occupation categories means that jobs that differ substantially from the perspective of their potential for automation are grouped together. For example, sales occupations will include both check-out operators – who are increasingly substituted by machines – and sellers expected to persuade customers to buy a product or service – a task that Autor et al. (2003) classify as non-routine.

The second classification is based on the Routine Task Intensity index provided in Table 1 of Goos et al. (2014). As their appendix explain, this uses the five original task measures from Autor et al. (2003) based on the US Dictionary of Occupational Titles from 1977 (see Appendix A.2 in Autor et al. 2003). They collapse the original five task measures into three task aggregates: the Manual task measure corresponds to the DOT variable for “eye-hand-foot coordination”; the Routine task measure is an average of “set limits, tolerances and standards” and “finger dexterity”; the Abstract task measure is the average of “direction control and planning” and “GED Math” – which measures mathematical and formal reasoning requirements. Appendix 1 in Autor et al. (2003) provides more details on these variables and offer some examples of tasks falling into each of these groups. While these indicators have been widely used in the literature, they are not exempt from criticism. For example, Green (2012) points out that in Autor et al. (2003) (and hence Goos et al. 2014) “adds and subtract 2-digit number” is part of the GED Math score which is classified as a non-routine task, in spite of being an easily codifiable task.

Because the variables in Autor et al. (2003) are available for US Census occupational codes, Goos et al (2014) have to implement several occupational conversions to be able to use them with ISCO88 codes: they convert the Census occupations into US SOC codes, then US SOC Codes into ISCO08 and finally ISCO08 into ISCO88 codes. Their paper reports the RTI index for 21 2-digit ISCO88 occupational codes.

Finally, I split occupations into routine vs non-routine depending on the level of their RTI index, using both employment-weighted and unweighted measures. For example, I classify as routine occupations the (employment-weighted) third with the highest RTI (as in Autor and Dorn 2013), or any occupation with an RTI higher than the unweighted average.

The third classification used in this paper uses task data from three waves of the British Skill Survey (1996, 2001, 2007). BSS contains 36 questions on activities workers perform on their job. Respondents are asked “We are interested in finding out what activities your job involves and how important these are” and they can rate the importance of each of the activities on a 5-point scale ranging from “essential” to “not at all important/not applicable”. As pointed out in Green (2012), it is not obvious how to split this long list into routine vs non-routine, i.e. more or less easy to automate. Table S5 reports the full list of tasks available and the classification adopted in Akcomak et al. (2016) which effectively leaves out the majority of tasks available.

I build a Routine Task Intensity index at the level of 2-digit ISCO88 codes to maximise comparability with Goos et al. (2014)^[[6]](#footnote-6)^. The RTI index is built following Autor and Dorn (2013) through the following steps:

1. I compute the occupation-level average score for each individual task over the three years (1997, 2001, 2006).
2. I divide the tasks into three groups: routine, service, cognitive (as in Table S5). For each occupation, I compute a score for each of these three groups of tasks.
3. Then for each occupation I compute a routine task intensity index as: ln(routine)-(ln(service)+ln(cognitive)).
4. This is then standardised across occupations.

I then identify the “routine occupations” based on the level of their RTI index, using both employment-weighted and unweighted measures, in the same fashion as for the indicator based on the RTI index provided by Goos et al. (2014).

The problem of classifying tasks (rather than occupations) as routine is one that affects both the approach based on the DOT definitions and the BSS data. The issue is further complicated by the fact that the range of tasks that can be automated changes over time as technology evolves and/or becomes more widespread. Arguably, for example, the task of “persuading/selling” might appear more easily subject to automation today than at the time of Autor et al. (2003) as indicated by the ever-more frequent use of automatically generated marketing messages based on online behaviour or spending habits traced by electronic payment systems.

A related issue, discussed in Autor (2013), is the mounting evidence that the task content of occupations changes over time, perhaps partly as a consequence of technological change. Akcomak et al. (2016) show substantial change in routine-intensity within occupations in the UK using BSS data. Hence differences in classifications based on task information from the late 1970s (such as the RTI index from Goos et al. 2014) and from the 1990s (such as the RTI index based on the BSS data) can reflect differences in changes in tasks within occupations. Following the prevailing approach in the literature, I focus on a comparison of static routine measures.

Table S6 shows the distribution of routine employment across the SOC90 major occupational groups based on these three classifications. For the two based on the RTI indexes, I consider both employment-weighted measures (i.e. the top employment-weighted third of occupations as in Autor and Dorn (2013)) and unweighted ones (i.e. occupations with an RTI larger than average) as indicated in the columns headings.

While clerical and secretarial occupations are consistently flagged as routine, there are notable differences across approaches for other occupational groups. Sales (as well as raft and machine operative) occupations, for example, are classified as routine by Acemoglu and Autor (2011), but less so under the weighted classifications based on the two RTI indexes. Most notably, “other (low skill) occupations”^[[7]](#footnote-7)^ are classified as routine by the AKR index, but not by either of the other two approaches.

| Table S5 - Classification of tasks from the British Skills Surveys into Routine and Non-routine (service or cognitive) | | |
| --- | --- | --- |
|  | Task description | Classification according to AKR 2013 |
| 1 | Paying close attention to detail | - |
| 2 | Dealing with people | service |
| 3 | Instruct, training or teaching people | cognitive |
| 4 | Making speeches or presentations | cognitive |
| 5 | Persuading or influencing | - |
| 6 | Selling a product or service | service |
| 7 | Counselling, advising or caring for customers | - |
| 8 | Working with a team of people | - |
| 9 | Listening carefully to colleagues | service |
| 10 | Physical Strength | - |
| 11 | Physical stamina | - |
| 12 | Skill and accuracy in using your hands or fingers | - |
| 13 | Knowledge of how to use or operate tools | - |
| 14 | Knowledge of particular products/services | service |
| 15 | Specialist knowledge or understanding | service |
| 16 | Knowledge of how your organisation works | - |
| 17 | Using a computer | - |
| 18 | Spotting problems or faults | routine |
| 19 | Working out causes of problems | - |
| 20 | Thinking of solutions to problems | cognitive |
| 21 | Analyse complex problems | cognitive |
| 22 | Checking there are no errors | routine |
| 23 | Noticing when there is a mistake | routine |
| 24 | Planning your own activities | - |
| 25 | Planning the activities of others | - |
| 26 | Organise your own time | - |
| 27 | Thinking ahead | - |
| 28 | Reading forms notices signs | - |
| 29 | Reading short docs, reports, letters | - |
| 30 | Reading long docs | - |
| 31 | Writing forms, notices, signs | - |
| 32 | Writing short documents | - |
| 33 | Writing long documents | - |
| 34 | Calculating (basic) | routine |
| 35 | Calculating using decimals, percentages, or fractions | routine |
| 36 | Using advanced maths or statistics | routine |

| **Table S6 -Distribution of routine employment across 1-digit occupations (SOC90) based on alternative classifications.** | | | | | | | | |
| --- | --- | --- | --- | --- | --- | --- | --- | --- |
| **SOC90 Major Occupational Group** | **1979 Total Employment Share** | **Routine employment share in 1979 based on alternative classifications:** | | | | | | |
|  |  | **AA 2011** | **GMS 2014 (a)** | | | **AKR 2013** | | |
|  |  |  | Top 30%, employment weighted | Top 50%, employment weighted | RTI above unweighted average | Top 30%, employment weighted | Top 50%, employment weighted | RTI above unweighted average |
| 1 Managers and Administrators | 8.10 | 0.00 | 0.00 | 0.00 | 0.00 | 0.00 | 0.20 | 0.20 |
| 2 Professional | 7.29 | 0.00 | 0.00 | 0.00 | 0.00 | 0.00 | 0.00 | 0.00 |
| 3 Associate professional and technical | 5.94 | 0.00 | 0.00 | 0.00 | 0.00 | 0.00 | 0.00 | 0.00 |
| 4 Clerical and secretarial | 18.86 | 18.86 | 18.78 | 18.78 | 18.78 | 15.59 | 15.59 | 18.78 |
| 5 Craft and related | 18.92 | 18.92 | 4.20 | 13.84 | 13.84 | 0.14 | 9.28 | 18.92 |
| 6 Personal and protective services | 7.68 | 0.00 | 0.00 | 0.00 | 0.83 | 0.83 | 0.83 | 0.83 |
| 7 Sales | 6.90 | 6.90 | 0.48 | 4.89 | 5.23 | 0.34 | 0.34 | 5.23 |
| 8 Plant and machine operatives | 14.80 | 14.80 | 6.38 | 9.46 | 9.46 | 12.83 | 14.58 | 14.65 |
| 9 Other occupations | 11.51 | 0.00 | 0.56 | 4.24 | 9.47 | 10.35 | 10.46 | 10.46 |
|  |  |  |  |  |  |  |  |  |
| Total share(b) | 100 | 59 | 30 | 51 | 58 | 40 | 51 | 69 |
| The details on each classification are provided in Section 0. AA: Acemoglu and Autor (2011). GMS: Goos et al. (2014). AKR: Akcomak et al. (2016).  (a): the RTI index of GMS is only available for 21 ISCO88 codes and therefore does not cover all occupations used in the analysis of this paper.  (b): the totals might not correspond to the column headings because ISCO88 2-digit codes correspond to large occupations making the running sum of employment by occupations a discrete rather than continuous variable. | | | | | | | | |

1. Or equivalently, compute the size of the destination occupation as a weighted average of the different origin occupations which “contributes” to it. [↑](#footnote-ref-1)
2. The LFS does not contain wage information till 1993. [↑](#footnote-ref-2)
3. Technically, some native workers who acquired their university-level qualification abroad might also have been affected. However, no clear discontinuity in the share of native workers reporting university education level is seen in 2011. [↑](#footnote-ref-3)
4. https://www.aeaweb.org/aer/app/10408/20111536_app.pdf [↑](#footnote-ref-4)
5. This is likely due to the stronger growth of education attainment among women than men. [↑](#footnote-ref-5)
6. ISCO88 codes are available in the BSS in 2001 and 2006. For 1997, I convert SOC90 (3 digit) ISCO88 (4 digits using the crosswalk available here: www.cf.ac.uk/socsi/CAMSI/occunits/uksoc90toisco88v1.sps. [↑](#footnote-ref-6)
7. This includes coal mine labourers, rail maintenance workers, refuse and salvage collectors, hospital, hotel and kitchen porters, lift and car park attendants, window cleaners and road sweepers, cleaners and domestics. [↑](#footnote-ref-7)
